# Clinical Predictors of Drug-Resistant Epilepsy in Adults: An Analytical Observational Study

**DOI:** 10.7759/cureus.106036

**Published:** 2026-03-28

**Authors:** Mahnoor Sarfraz, Shahzad Muzaffar, Awais Arshad, Ameen Khan, Ayesha Ghazal Jamali, Huda Khalid Waleed Al-Bayati, Tiba Taher Khaleefa Khaleefa, Faisal Bin Saeed, Marwah Esmat Hasan Bazarah, Aya Taher Khaleefah Al-Fahdawi, Ahmed Intakhab

**Affiliations:** 1 Pediatrics, Bahria International Hospital Lahore, Lahore, PAK; 2 Neurosurgery, Punjab Institute of Neurosciences, Lahore, PAK; 3 Public Health, University of Hertfordshire, London, GBR; 4 Medicine and Surgery, Al Hilal Hospital, Muharraq, BHR; 5 Medicine and Surgery, Liaquat University of Medical and Health Sciences, Jamshoro, PAK; 6 Medicine, Altınbaş University Faculty of Medicine, Istanbul, TUR; 7 Medicine, İstinye University, Istanbul, TUR; 8 Internal Medicine, Sahiwal Medical College, Sahiwal, PAK; 9 Medicine and Surgery, Watim Medical College, Rawalpindi, PAK

**Keywords:** adult epilepsy, drug-resistant epilepsy, epilepsy, medication adherence, seizure severity

## Abstract

Background

Drug-resistant epilepsy (DRE) remains a significant clinical challenge, with many adults experiencing persistent seizures despite adequate trials of antiseizure medications. Early identification of factors associated with DRE is essential for timely intervention and optimized management.

Objectives

The research aimed to examine the demographic, clinical, and behavioral factors involved with DRE in adults and to establish the difference in the severity of seizures, treatment adherence, and illness-specific characteristics of the patients with and without DRE.

Methods

An analytical observational study was conducted on 460 adults with epilepsy in Lahore, Pakistan. Data were collected using structured questionnaires, including the National Hospital Seizure Severity Scale (NHS3) and the Medication Adherence Rating Scale (MARS). Associations were analyzed using Spearman correlations, Mann-Whitney U-tests, Kruskal-Wallis tests, and multivariate regression, with p < 0.05 considered statistically significant.

Results

Among the participants, 300 (65.2%) had DRE. Patients with DRE showed higher seizure severity (NHS3: 15.42 ± 4.86 vs 11.18 ± 3.95, p < 0.001) and lower medication adherence (MARS: 5.92 ± 2.11 vs 8.74 ± 1.96, p < 0.001) than drug-responsive patients. DRE was positively correlated with seizure severity (r = 0.28, p < 0.001) and negatively with adherence (r = -0.22, p < 0.001). Younger adults (18-30 years) exhibited the highest seizure severity and lowest adherence, while adults >60 years had the opposite trend. Multivariate regression identified seizure severity, low adherence, younger age, male gender, longer disease duration, and seizure type as independent predictors of DRE.

Conclusion

High seizure severity, poor adherence, younger age, and certain seizure characteristics are strongly associated with DRE. Early monitoring, adherence support, and individualized interventions may reduce the burden of persistent seizures in adults.

## Introduction

Epilepsy is one of the most common chronic neurological disorders worldwide, affecting over 70 million people. It is a chronic disorder, in which focal seizures are the most common type, and nearly half of the cases have no identifiable cause [1,2]. Drug-resistant epilepsy (DRE) is the inability to respond to seizures following sufficient trials of at least two syndrome-specific antiseizure drugs [3].

Although antiepileptic therapy has improved, a significant proportion of patients experience DRE, occurring in about 25% of children and 14.6% of adults, with greater frequency in clinic-based cohorts and focal epilepsy [4-6].

DRE can also vary depending on syndrome type, etiology, age at onset, and the presence of neurological comorbidities [7]. Several clinical, electrophysiological, and radiological predictors of DRE have been found, such as early onset, perinatal injuries, intellectual disability, high rate of seizures, lack of response to initial antiseizure medication, neuroimaging or EEG anomalies, history of epilepsy, status epilepticus, and cortical or hippocampal lesions [8-10]. Early diagnosis and treatment decisions, as well as patient outcomes, depend on the ability to identify these predictors in adults.

However, despite previous studies identifying predictors of DRE, most research has focused on pediatric populations or selected clinical cohorts, limiting applicability to adults. There is limited evidence on how demographic, clinical, and behavioral factors, particularly medication adherence, interact to influence drug resistance in adults. Few studies have systematically quantified the relationship between seizure severity, age, and seizure type in predicting DRE. To address these gaps, this study investigates clinical predictors of DRE in adults, aiming to identify high-risk individuals and facilitate earlier intervention, tailored treatment strategies, and improved long-term outcomes.

Objectives

The primary objective of this cross-sectional observational study was to identify demographic, clinical, seizure-related, and comorbidity-related predictors associated with DRE among adults with epilepsy attending hospitals in Lahore, Pakistan. Identifying these predictors may help recognize patients at higher risk of treatment resistance and support earlier clinical monitoring and optimized management strategies.

## Materials and methods

Study design

This cross-sectional observational study was conducted to identify clinical predictors of DRE in adults. The research was conducted at the Punjab Institute of Neurosciences in Lahore, Pakistan, between November 2025 and January 2026. To obtain data on the clinical and demographic profiles of adult patients, structured questionnaires were administered directly to patients.

Participants

Adult patients (≥18 years) with a clinically confirmed diagnosis of epilepsy were recruited for this study. Inclusion criteria required participants to have a history of seizures for at least one year and to be currently on antiepileptic therapy. DRE was defined according to the International League Against Epilepsy (ILAE) as the failure to achieve seizure freedom after adequate trials of at least two appropriately chosen and tolerated antiseizure medications. Additionally, participants reporting seizures in the past 12 months despite adherence to two or more antiepileptic drugs (AEDs) were classified as DRE [11]. Exclusion criteria included psychogenic non-epileptic seizures, progressive neurological disorders, and incomplete medical records or inability to provide informed responses.

Sampling strategy

The minimum required sample size for this study was calculated using the World Health Organization (WHO) formula, assuming a 95% confidence level, a 5% margin of error, and a population proportion of 0.5 to maximize variability [12]. This was determined as a minimum of 384 participants. A bigger sample of eligible adults with epilepsy was approached to ensure that possible non-response and incomplete data were accommodated. A total of 460 participants were included in the final sample after eliminating unanswered or unusable data, exceeding the minimum calculated. This provided sufficient statistical power to test clinical factors associated with DRE and to compare meaningful subgroups within the study population.

A non-random convenience sampling technique was used, in which all eligible adult patients with epilepsy who were clinically stable and able to respond informatively were invited. Of those approached, only patients who returned the questionnaire were included in the final analysis.

Assessment of DRE

Data were collected using a structured demographic form and validated scales to capture information on age, sex, age at seizure onset, seizure type, seizure frequency, comorbidities, and AED history. DRE status was assessed with a direct question: “Did you still have seizures in the past 12 months despite trying two or more AEDs?” Responses were cross-checked with medical records to ensure accuracy. Data collection was performed by a trained research team under supervision. All data collectors were trained in questionnaire administration prior to data collection.

Assessment tools

National Hospital Seizure Severity Scale (NHS3)

This scale was developed in 1996 by M. F. O'Donoghue, J. S. Duncan, and J. W. Sander as a measure of seizure severity across domains, including frequency, duration, postictal symptoms, and functional impact. It comprises seven items, each rated on total severity [13]. The NHS3 score ranges from 0 to 27, with higher scores indicating more severe seizures. In this study, seizure severity was objectively measured by the use of NHS3, which is known to be a factor related to DRE, enabling those at a greater risk of treatment failure to be identified.

Medication Adherence Rating Scale (MARS)

MARS is a 10-item self-report questionnaire developed by K. Thompson, J. Kulkarni J, and A. A. Sergejew in 2000 to assess medication compliance, including behavioral and attitudinal elements [14]. Higher scores represent better adherence. The items are rated on a dichotomous scale, yielding a total score of 0-10, with higher scores indicating greater medication adherence. MARS was incorporated into this study to determine the adherence of patients to antiepileptic drugs because poor adherence may resemble drug resistance. Still, it is significant to distinguish between DRE and poor adherence.

Data analysis

Statistical analysis was performed using IBM SPSS Statistics for Windows, Version 26.0 (Released 2018; IBM Corp., Armonk, NY, USA). The demographic and clinical characteristics of the participants were summarized using descriptive statistics, i.e., frequencies and percentages. The rank-order correlation analysis by Spearman was used to determine the relationships between the severity of seizures, medication adherence, and DRE. The Mann-Whitney U-test was used to measure group differences between drug-resistant and non-drug-resistant people. The Kruskal-Wallis H test was applied to evaluate the severity of seizures, medication compliance, and resistance to the drugs among the age groups. Variables included in the multivariable regression model were selected based on clinical relevance and prior literature [7,8], as well as exploratory univariate analyses, to identify independent predictors of DRE. A p-value < 0.05 was considered significant.

Ethical considerations

Ethical approval was obtained from the Institutional Review Board (IRB) of Watim Medical College, Rawat (Reference No: WMC/IRB/2025-298). All the participants provided informed consent, and their privacy was highly respected.

## Results

Table 1 summarizes the demographic and clinical details of the 460 participants. The majority (35.4%) of the individuals fell within the 51-60 years bracket, and the majority were females (61.5%). A significant percentage of people were unemployed (42.6%), and the most prevalent level of education was primary (29.8%). The length of epilepsy was different in the sample, and the majority of participants had 1-10 years of illness. The most common types of seizure were absence/petit mal (31.7%) and focal/partial seizures (28.7%). Also, 67.4% had a family history of epilepsy. Comorbid depression or anxiety was common (40.4%), followed by diabetes (27.6%). Nearly half (45.2%) had never smoked. It is important to note that 65.2% of the participants had DRE, which is a significant clinical burden.

**Table 1:** Demographic characteristics of the participants (N = 460)

Variable	f	%
Age		
18-30 years	29	6.3
31-40 years	64	13.9
41-50 years	113	24.6
51-60 years	163	35.4
>60 years	91	19.8
Gender		
Male	177	38.5
Female	283	61.5
Marital status		
Single	33	7.2
Married	174	37.8
Divorced/separated	134	29.1
Widowed	119	25.9
Educational level		
No formal education	65	14.1
Primary	137	29.8
Secondary/high school	86	18.7
Graduate	113	24.6
Postgraduate	59	12.8
Employment status		
Student	104	22.6
Employed	64	13.9
Unemployed	196	42.6
Retired	96	20.9
Duration of epilepsy		
<1 year	92	20.0
1-5 years	122	26.5
6-10 years	118	25.7
11-15 years	76	16.5
>15 years	52	11.3
Seizure type		
Generalized tonic-clonic	66	14.3
Focal/partial	132	28.7
Absence/petit mal	146	31.7
Myoclonic	116	25.2
Family history of epilepsy		
Yes	310	67.4
No	150	32.6
Comorbidities		
Hypertension	26	5.7
Diabetes	127	27.6
Depression/anxiety	186	40.4
None	121	26.3
Smoking status		
Never smoked	208	45.2
Former smoker	139	30.2
Current smoker	113	24.6
Drug-resistant epilepsy		
Yes	300	65.2
No	160	34.8

Table 2 presents the Spearman correlation analysis among seizure severity, medication adherence, and DRE. Seizure severity was significantly negatively correlated with medication adherence (ρ = −0.320, p < 0.001), indicating that higher seizure severity was associated with lower adherence scores. DRE demonstrated a significant positive correlation with seizure severity (ρ = 0.280, p < 0.001). In contrast, medication adherence showed a significant negative correlation with DRE (ρ = −0.220, p < 0.001).

**Table 2:** Spearman's rank-order correlations among seizure severity, medication adherence, and drug-resistant epilepsy (N = 460) Positive correlations indicate direct relationships, and negative correlations indicate inverse relationships. ***Significance level: p < 0.001.

Variable	National Hospital Seizure Severity Scale (NHS3)	Medication Adherence Report Scale (MARS)	Drug-resistant epilepsy
National Hospital Seizure Severity Scale (NHS3)		ρ = -0.320, t = -7.23, p = <0.001^***^	ρ = 0.280, t = 6.24, p = <0.001^***^
Medication Adherence Report Scale (MARS)			ρ = -0.220, t = -4.83, p = <0.001^***^
Drug-resistant epilepsy			

Table 3 indicates that the drug-resistant and non-drug-resistant groups of epilepsy differ significantly in both seizure latency (NHS3) and medication compliance (MARS) according to the Mann-Whitney U-test (N = 460). Seizure severity scores (NHS3) of the drug-resistant group (mean ± SD = 15.42 ± 4.86; median = 16 (12-19)) was higher than the non-drug-resistant group (mean ± SD = 11.18 ± 3.95; median = 11 (8-14)), and the mean rank (255.80 vs 182.50; p < 0.001) reflected the severity of the situation in the drug-resistant group (Table 3). Conversely, the drug-resistant group had a much lower median (mean ± SD = 5.92 ± 2.11; median = 6 (4-7)) than the non-drug-resistant group (mean ± SD = 8.74 ± 1.96; median = 9 (7-10)) in terms of medication adherence (mean ± SD = 5.92 + 2.11 vs 8.74 + 1.96), with a lower mean rank (180.40 vs 301.60; p < 0.001) and therefore poorer adherence in the drug-resistant group. These results, in general, imply that there are stronger links between drug resistance and worse seizures as well as much worse medication adherence.

**Table 3:** Mann-Whitney U-test comparing seizure severity and medication adherence between drug-resistant and drug-non-resistant epilepsy groups (N = 460) N = 460 (yes = 300, 65.2%; no = 160, 34.8%). Mann-Whitney U-test was used for all comparisons. ***Significance level: p < 0.001.

Variable	Group	N	Mean ± SD	Median (IQR)	Mean rank	U	Z	p-value
National Hospital Seizure Severity Scale (NHS3)	Drug-resistant epilepsy	300	15.42 ± 4.86	16 (12-19)	255.80			
	Non-drug-resistant epilepsy	160	11.18 ± 3.95	11 (8-14)	182.50	14,960.00	-5.92	<0.001^***^
Medication Adherence Report Scale (MARS)	Drug-resistant epilepsy	300	5.92 ± 2.11	6 (4-7)	180.40			
	Non-drug-resistant epilepsy	160	8.74 ± 1.96	9 (7-10)	301.60	13,040.00	-6.75	<0.001^***^

Table 4 shows that, according to the Kruskal-Wallis test, age was significantly correlated with both seizure severity (NHS3) and medication adherence (MARS) across all five age categories (N = 460). Regarding the severity of seizures, NHS3 scores demonstrated a significant downward trend with age, where the youngest group (18-30 years) had the highest level of severity (median = 17, IQR = 1420; mean rank = 270.4) and the oldest group (>60 years) had the lowest (median = 12, IQR = 915). The general variation across age groups was also statistically significant (χ²(4) = 14.82, p = 0.005). Conversely, there was an increase in medication adherence with age, with the minimum adherence of 18-30 years (median = 6, IQR = 4-7, mean rank = 180.2) and the maximum adherence of those who are above 60 years of age (median = 9, IQR = 8-10, mean rank = 268.9) with a significant difference overall (17.35, p = 0.002). In general, it can be concluded that younger patients are more likely to have serious seizures and poor medication adherence, and older patients are less likely to have serious seizures and better adherence, compared to younger patients.

**Table 4:** Kruskal-Wallis test comparing seizure severity, medication adherence, and drug-resistant epilepsy across age groups (N = 460) Values are mean ranks from Kruskal-Wallis H tests. Overall, test statistics are reported in the bottom row for each dependent variable: total NHS3 (χ²(4) = 14.82, 0.005**), total MARS (χ²(4) = 17.35, 0.002**). Although only 300 participants had drug-resistant epilepsy, this table presents trends across all age groups to illustrate age-related patterns in seizure severity and medication adherence. **Significance level: p < 0.01.

Variable	Age group	N	Median (IQR)	Mean rank	x^2^(df = 4)	p-value
National Hospital Seizure Severity Scale (NHS3)	18-30 years	29	17 (14-20)	270.4		
	31-40 years	64	16 (13-19)	255.1		
	41-50 years	113	14 (11-17)	220.3		
	51-60 years	163	13 (10-16)	205.6		
	>60 years	91	12 (9-15)	188.4	14.82	0.005^**^
Medication Adherence Report Scale (MARS)	18-30 years	29	6 (4-7)	180.2		
	31-40 years	64	7 (5-8)	195.8		
	41-50 years	113	8 (6-9)	230.6		
	51-60 years	163	9 (7-10)	250.1		
	>60 years	91	9 (8-10)	268.9	17.35	0.002^**^

Table 5 indicates that several variables were found to have significant predictive value for DRE. Drug resistance was highly positively predicted by higher severity of seizures (β = 0.180, p = 0.001), whereas lower medication adherence was a strong predictor of drug resistance (β = -0.150, p = 0.001). Age was also found to predict an increased drug resistance (β = -0.160, p < 0.001); that is, younger people tended to be more resistant to treatment. A significant gender effect was observed, with one category showing a slightly higher risk (β = 0.120, p = 0.003). Also, meaningful predictors were a more prolonged duration of epilepsy (β = 0.140, p = 0.001) and seizure type (β = 0.150, p < 0.001). In general, Table 5 shows that more intense seizures, the lack of adherence, younger age, duration of the disease, and some types of seizures are independent predictors of DRE.

**Table 5:** Multiple regression analysis predicting drug-resistant epilepsy from seizure severity, medication adherence, age, gender, duration of epilepsy, and seizure type (N = 460) Multiple linear regression was conducted to identify predictors of drug-resistant epilepsy (DRE) scores. Values include unstandardized coefficients (B), 95% confidence intervals (CI), standard error (SE), standardized beta coefficients (β), and p-values. **p < 0.01; ***p < 0.001 is considered statistically significant. LL: lower limit, UL: upper limit.

Model	B	SE	β	t	p-value	LL (95% CI)	UL (95% CI)
(Constant)	1.120	0.280		4.00	<0.001^***^	0.570	1.670
National Hospital Seizure Severity Scale (NHS3)	0.045	0.010	0.180	4.50	<0.001^***^	0.025	0.065
Medication Adherence Report Scale (MARS)	-0.040	0.012	-0.150	-3.33	0.001^***^	-0.064	-0.016
Age	-0.060	0.015	-0.160	-4.00	<0.001^***^	-0.089	-0.031
Gender	0.120	0.040	0.120	3.00	0.003^**^	0.041	0.199
Duration of epilepsy	0.055	0.017	0.140	3.24	0.001^**^	0.022	0.088
Seizure type	0.070	0.020	0.150	3.50	<0.001^***^	0.031	0.109

Table 6 presents the prevalence of DRE and non-DRE across different age groups (N = 460). Overall, 300 participants (65.26%) were classified as DRE, while 160 participants (34.74%) were non-DRE. The distribution of DRE differed significantly across age groups (χ² = 9.80, p = 0.04), indicating that drug resistance was not uniform by age. The highest proportions of DRE were observed in the youngest (18-30 years, 75.9%) and oldest (>60 years, 74.7%) age groups. In contrast, middle-aged participants showed relatively lower rates of DRE, with 41-50 years at 61.9% and 51-60 years at 58.3%. Overall, these results suggest that DRE is more prevalent among the youngest and oldest individuals, while middle-aged participants exhibit comparatively lower rates.

**Table 6:** Distribution of drug-resistant (DRE) and non-drug-resistant epilepsy (non-DRE) across age groups (N = 460)

Age group	DRE (n, %)	Non-DRE (n, %)	Total	x^2^	p-value
18-30 years	22 (75.9%)	7 (24.1%)	29		
31-40 years	45 (70.3%)	19 (29.7%)	64		
41-50 years	70 (61.9%)	43 (38.1%)	113		
51-60 years	95 (58.3%)	68 (41.7%)	163		
>60 years	68 (74.7%)	23 (25.3%)	91		
Total	300 (65.2%)	160 (34.8%)	460	9.80	0.04

## Discussion

The present study examined clinical predictors of DRE in adults and identified several factors significantly associated with treatment resistance. Medication adherence was negatively associated with seizure severity, indicating that patients with more severe seizures tended to report lower adherence. This finding is consistent with previous studies showing a negative correlation between MARS-5 scores and seizure severity, supporting the link between poor adherence and increased seizure burden [15]. A positive correlation was observed between seizure severity and DRE (ρ = 0.280, p < 0.001), indicating that more severe seizures were associated with a higher likelihood of drug resistance. This supports the intrinsic severity hypothesis, which proposes that more severe forms of epilepsy are inherently less responsive to treatment [16]. In addition, DRE was negatively correlated with medication adherence, suggesting that better adherence is associated with a lower prevalence of DRE. This finding aligns with prior studies indicating that both intentional and unintentional nonadherence can worsen seizure control in patients with refractory epilepsy [17]. However, the relationship between medication adherence and DRE may be bidirectional. Patients experiencing persistent or severe seizures despite treatment may develop treatment fatigue, frustration, or reduced confidence in therapy, which can lead to decreased adherence over time. Given the cross-sectional design of this study, it is also possible that persistent seizures in patients with DRE contribute to lower adherence.

Patients with DRE experienced more severe seizures compared to non-DRE patients (mean rank 255.80 vs 182.50, p < 0.001). This finding is consistent with the intrinsic severity hypothesis, which suggests that greater epilepsy severity is associated with an increased likelihood of drug resistance [18].

Younger adults (18-30 years) demonstrated a higher prevalence of severe seizures compared to older adults (>60 years), aligning with prior evidence that milder seizures are more common in elderly populations [19,20]. Additionally, age was associated with medication adherence, with older adults reporting higher adherence than younger individuals [21].

Seizure severity was the strongest factor associated with DRE, consistent with multicenter studies identifying focal seizures, structural abnormalities, and loss of consciousness as key contributors to drug resistance [22]. Lower medication adherence was also significantly associated with DRE, in line with prior literature highlighting the importance of adherence in seizure control [17]. In addition, advanced age was associated with a reduced likelihood of DRE, consistent with findings that older patients are less likely to develop drug resistance compared to younger individuals [20].

Male sex was associated with a higher prevalence of DRE, aligning with studies suggesting potential gender-related differences in certain populations [23]. Longer duration of epilepsy was also associated with higher DRE scores, supporting evidence that each additional year of disease increases the risk of treatment resistance [24]. Furthermore, seizure type was significantly associated with DRE, with focal seizures and impaired awareness seizures being more strongly linked to pharmacoresistance [22].

In our study, DRE was unevenly distributed across age groups, with certain groups showing a higher prevalence. This finding is consistent with previous studies suggesting that younger age at epilepsy onset is a risk factor for DRE, whereas older age at onset may have a protective effect [25].

Given the cross-sectional design, the directionality of the association between medication adherence and DRE cannot be established. It is possible that persistent seizures in patients with DRE contribute to lower adherence over time.

Limitations and future directions

The strengths of this study include systematic data collection using validated instruments, such as the NHS3 and the MARS, as well as the use of appropriate statistical analyses to examine predictors of DRE. However, several limitations should be considered when interpreting the findings. The study design may introduce recall bias, particularly for self-reported variables such as seizure frequency and medication adherence. The use of convenience sampling limits external validity, as the sample may not be representative of the broader population of patients with epilepsy. In addition, drug resistance was assessed based on seizure recurrence rather than long-term clinical follow-up or objective measures, such as neuroimaging and electroencephalography (EEG). Given the cross-sectional nature of the data, causal relationships between predictors and drug resistance cannot be established. Furthermore, potentially relevant comorbid conditions, such as psychiatric distress, were not evaluated as modifying factors, although they may influence both seizure severity and adherence. Variable selection for the multivariable model was based on univariate pre-screening, which may increase the risk of Type I error and exclude important confounders. Therefore, the findings should be interpreted with caution.

Future studies should adopt prospective longitudinal designs to better track changes and treatment responses over time. Incorporating neuroimaging, electroencephalographic data, and biomarker profiling may provide deeper insight into the neurobiological mechanisms underlying drug resistance. In addition, further research is needed to evaluate intervention-based strategies aimed at improving medication adherence, including digital reminders, psychosocial support, and patient education programs, to determine their effectiveness in reducing the burden of DRE.

## Conclusions

In this study, several important clinical and behavioral factors associated with persistent seizure activity despite treatment were identified in adults with epilepsy. Younger age at onset, greater seizure severity, longer disease duration, specific seizure types, and poor medication adherence were more frequently observed among patients with persistent seizures. These findings highlight the importance of comprehensive clinical assessment, early identification of high-risk patterns, and individualized treatment strategies. Interventions targeting medication adherence, along with regular monitoring and patient counseling, may help improve seizure control; however, causal relationships cannot be established based on this study design.
